# Transcatheter aortic valve implantation for aortic stenosis in high surgical risk patients: A systematic review and meta-analysis

**DOI:** 10.1371/journal.pone.0196877

**Published:** 2018-05-10

**Authors:** Zulian Liu, Elaine Kidney, Danai Bem, George Bramley, Susan Bayliss, Mark A. de Belder, Carole Cummins, Rui Duarte

**Affiliations:** 1 Nuffield Department of Population Health, University of Oxford, Oxford, United Kingdom; 2 Institute of Applied Health Research, University of Birmingham, Birmingham, United Kingdom; 3 The James Cook University Hospital, Middlesbrough, United Kingdom; 4 Liverpool Reviews and Implementation Group, University of Liverpool, Liverpool, United Kingdom; East Tennessee State University, UNITED STATES

## Abstract

**Background:**

Symptomatic aortic stenosis has a poor prognosis. Many patients are considered inoperable or at high surgical risk for surgical aortic valve replacement (SAVR), reflecting their age, comorbidities and frailty. The clinical effectiveness and safety of TAVI have not been reviewed systematically for these high levels of surgical risk. This systematic review compares mortality and other important clinical outcomes up to 5 years post treatment following TAVI or other treatment in these risk groups.

**Methods:**

A systematic review protocol was registered on the PROSPERO database (CRD42016048396). The Cochrane Library, Centre for Reviews and Dissemination Databases, MEDLINE, EMBASE, and ZETOC were searched from January 2002 to August 2016. Clinical trials or matched studies comparing TAVI with other treatments for AS in patients surgically inoperable or operable at a high risk were included. Data extraction and quality assessment were conducted by two reviewers. Data were pooled using random-effects meta-analysis. The main outcomes were all-cause mortality, efficacy and major complications.

**Results:**

Three good quality randomised controlled trials (RCTs) were included. Patients’ mean age ranged from 83–85 years, around half were female and New York Heart Association (NYHA) functional class III or IV ranged from 83.8% to 94.2% with frequent comorbidities. In 358 surgically inoperable patients from one RCT, TAVI was superior to medical therapy for all-cause mortality at 1 year (hazard ratio (HR) 0.58, 95% confidence interval (CI) 0.36−0.92), 2 years (HR 0.50, 95% CI 0.39−0.65), 3 years (HR 0.53, 95% CI 0.41to 0.68) and 5 years (HR 0.50, 95% CI 0.39−0.65), and NYHA class III or IV at 2 years (TAVI 16.8% (16/95), medical therapy 57.5% (23/40), p<0.001), quality of life and re-hospitalisation. TAVI had higher risks of major bleeding up to 1 year, of stroke up to 3 years (at one year 11.2% versus 5.5%, p = .06; HR at 2 years 2.79, 95% CI 1.25−6.22; HR at 3 years 2.81; 95% CI 1.26−6.26) and of major vascular complication at 3 years (HR 8.27, 95% CI 2.92−23.44). Using the GRADE tool, this evidence was considered to be of moderate quality. In a meta-analysis including 1,494 high risk surgically operable patients from two non-inferiority RCTs TAVI showed no significant differences from SAVR in all-cause mortality at two years (HR 1.03, 95% CI 0.82−1.29) and up to 5 years (HR 0.83, 95% CI 0.83−1.12). There were no statistically significant differences in major vascular complications and myocardial infarction at any time point, discrepant results for major bleeding on variable definitions and no differences in stroke rate at any time point. Using the GRADE tool, this evidence was considered of low quality.

**Conclusions:**

Symptomatic aortic stenosis can be lethal without intervention but surgical resection is contraindicated for some patients and high risk for others. We found that all-cause mortality up to 5 years of follow-up did not differ significantly between TAVI and SAVR in patients surgically operable at a high risk, but favoured TAVI over medical therapy in patients surgically inoperable. TAVI is a viable life-extending treatment option in these surgical high risk groups.

## Introduction

Aortic stenosis causes impaired outflow of blood from the heart and is usually progressive. It increases cardiac workload and leads to left ventricular hypertrophy and heart failure. Symptomatic aortic stenosis with angina, syncope or heart failure is associated with an annual mortality rate of around 25%, and, without mechanical relief of the obstruction to the aortic outflow, has a very poor prognosis.[1]

Surgical aortic valve replacement (SAVR) with an artificial prosthesis is the conventional treatment for severe aortic valve stenosis. However, some patients may not be suitable to receive SAVR because of medical comorbidities (most patients are aged 75 or older) or because of technical considerations (for example if the patient has a calcified aorta or scarring from previous cardiac surgery). For these patients, conventional treatment has been optimal medical care, which however can only ease some symptoms.[1]

Transcatheter aortic valve implantation (TAVI) has been used for over a decade as a less invasive option for those who cannot undergo SAVR due to high risk of surgical complications.[2,3] Following continuous advancements in TAVI technology with the aim of reducing complications, the use of TAVI has been extended to patients for whom SAVR is considered suitable but poses a high risk and also intermediate and lower risk patient populations, including younger patients with fewer comorbidities.[3]

The clinical effectiveness and safety of TAVI has been evaluated in groups of patients with different surgical risks. Systematic reviews have assessed the evidence for the overall patient population [4] and for the patient population with lower surgical risk.[5,6] However, the evidence on the use of TAVI in surgically inoperable or high risk patients has not been reviewed systematically. As medical treatments are not effective, the aim of this systematic review is to assess the clinical effectiveness and safety of TAVI for patients with severe aortic stenosis for whom SAVR is not an option or presents a high risk and for whom if effective TAVI might offer an improved prognosis.

## Methods and materials

We carried out a systematic review on the effectiveness and safety of TAVI compared with other treatments for severe aortic stenosis in patients surgically inoperable and in patients surgically operable at a high risk of complications. This systematic review was part of a wider risk stratified systematic review of evidence on the use of TAVI in patients with severe aortic stenosis, which was carried out to support an update of the Interventional Procedures Guidance on TAVI issued by the UK National Institute for Health and Care Excellence (NICE).[6]

A protocol was developed for the wider systematic review according to the Preferred Items for Systematic Reviews and Meta-Analysis for systematic review Protocols (PRISMA-P) recommendations [7] and was registered with PROSPERO (CRD42016048396). The current systematic review is reported in accordance with the PRISMA recommendations for reporting systematic reviews (S1 Table).[8]

### Data sources and searches

Electronic databases including the Cochrane Library (CDSR, DARE, HTA and CENTRAL), Centre for Reviews and Dissemination Databases (DARE, NHS EED and HTA), MEDLINE, MEDLINE in Process, EMBASE, ZETOC and PubMed were searched from January 2002 to August 2016. Comprehensive search strategies were developed using a combination of both index and free text terms. Reference lists of relevant papers and systematic reviews were hand-searched, and trial registries, including ClinicalTrials.gov and WHO ICTRP were also searched. Relevant websites were searched and experts contacted (see S2 Table).

### Study selection

Two reviewers independently screened the titles and abstracts of all citations (R.D., Z.L., E.K., G.B., D.B.). The full-text of potentially relevant studies was retrieved for further independent assessment by two reviewers (R.D., Z.L., E.K., G.B., D.B.). Any disagreements were resolved by discussion and consensus between the reviewers or consultation with a third reviewer (R.D., Z.L., E.K., G.B., D.B.) if consensus was not reached.

Published English-language literature including randomised controlled trials (RCTs), non-randomised trials, and propensity score matched studies were included if they reported clinical data of TAVI versus other treatments for severe aortic stenosis in patients considered to be surgically inoperable or in patients for whom SAVR was considered suitable but posing a high risk.

Studies were excluded where: (i) the patient surgical risk level was unclear, or included patients of a range of different surgical risk levels (such as high, intermediate or low risk) with data not presented separately by surgical risk level; (ii) the focus was on the impact of ancillary variations of the TAVI procedure (such as types of anaesthetic; imaging examination or guidance; learning curve) rather than different types of devices and different implantation techniques that are directly related to TAVI valves, delivery systems or equipment, and implantation technique including delivery route and positioning; (iii) the intervention was transcatheter valve-in-valve implantation for aortic bioprosthetic valve dysfunction; (iv) TAVI in combination with any other surgical cardiac procedure, or the comparator was SAVR in combination with any other surgical cardiac procedure; (v) surrogate outcomes (such as biomarkers or platelet volume) were reported rather than clinical outcomes; or (vi) only an abstract was available.

### Data extraction and quality assessment

For each included study, data were extracted by one reviewer and checked for accuracy and completeness by a second reviewer (E.K., G.B., Z.L., D.B., R.D.). Any disagreements were resolved by discussion and if necessary consultation with a third reviewer (E.K., G.B., Z.L., D.B., R.D.).

For survival data, the hazard ratio (HR) and its variance or other data that could be used to calculate HR and variance according to the methods described by Tierney et al. [9] were extracted from the reports with the longest follow up times.

The Cochrane Collaboration’s risk of bias tool was used to assess the quality of RCTs.[10] Quality assessment was conducted by one reviewer (R.D.) and checked by a second reviewer (Z.L.). Disagreements were resolved by discussion and consensus and if necessary consultation with a third reviewer. GRADE framework was employed to describe the quality of key outcomes and the overall strength of the supporting evidence from the RCTs.[11]

### Data synthesis and analysis

Main outcomes of interest of the review were all-cause mortality and cardiac mortality. Secondary outcomes included: (i) clinical efficacy outcomes, including cardiac function/New York Heart Association (NYHA) heart failure class, quality of life (QoL), technically successful valve implantation, and reduction of symptoms; (ii) hemodynamic performance data in terms of occurrence of aortic regurgitation; (iii) safety outcomes of complications and adverse events including perioperative mortality, stroke, major bleeding, acute kidney injury, myocardial infarction, atrial fibrillation, major vascular complication, prosthesis-patient mismatch, permanent pacemaker implantation, hospital readmission, and short and long term valve function/durability.

Studies were categorised into two groups according to surgical risk level of the patients in the studies: (i) surgically inoperable; (ii) surgically operable at a high risk.

A narrative synthesis was employed where data were insufficient or where it was inappropriate to combine studies statistically. Where possible, meta-analyses were carried out in RevMan 5.3 using random effects model. Intention-to-treat (ITT) methods according to the initial treatment assignment were adopted where appropriate. Dichotomous data were expressed as risk ratio (RR) with 95% confidence interval (CI). For survival data HRs were pooled using the generic inverse variance method with a fixed effect model. Continuous data were analysed by calculating the weighted mean difference (WMD) between groups and the corresponding 95% CI. Heterogeneity was explored using the I^2^ statistic and chi-square test of heterogeneity.[10]

As the number of studies included in the meta-analyses conducted was limited, funnel plot asymmetry testing for publication bias, sensitivity analyses exploring the robustness of the meta-analyses and meta-regression exploring study-level predictors were not conducted.

## Results

**Fig 1:**
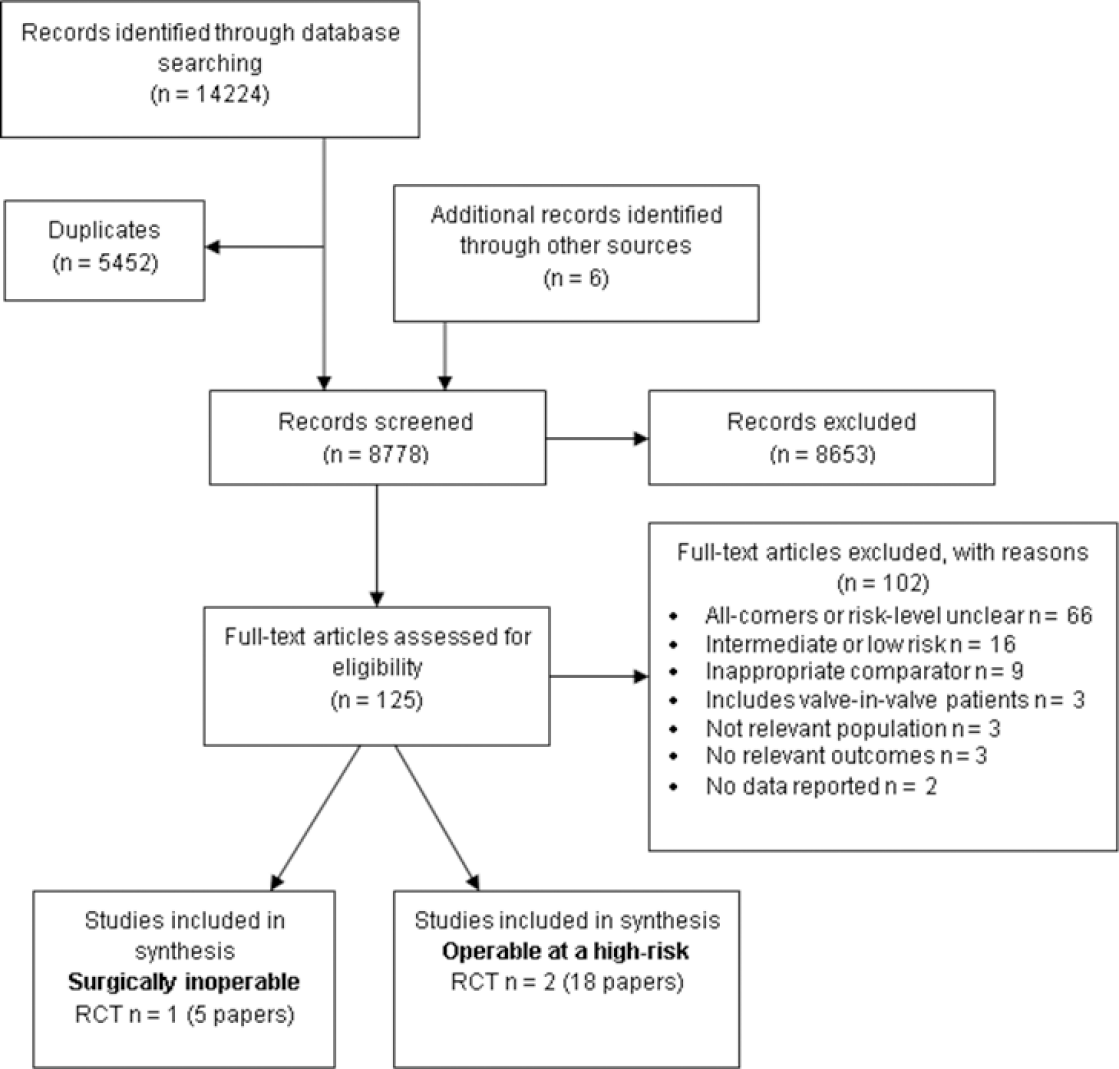
Flow diagram presenting the search and selection processes.

The searches resulted in 14,224 citations. Following screening of titles and abstracts, 125 papers were retrieved for detailed assessment of eligibility, of which 23 met the inclusion criteria (Fig 1). One RCT (PARTNER 1B) reported in 5 papers [12–16] in surgically inoperable patients, and 2 RCTs (PARTNER 1A and US CoreValve) reported in 18 papers [17–34] in patients surgically operable at a high risk, were included in the analyses (Fig 1).

A number of propensity-score matched studies were identified comparing TAVI with SAVR, but were excluded because the surgical risk level of the patients receiving TAVI was unclear.

In the PARTNER 1B trial TAVI was compared with usual care (medical therapy) in 358 patients considered surgically inoperable (Table 1). A balloon expanding heart-valve and the transfemoral (TF) route were used for TAVI. Usual care comprised balloon aortic valvuloplasty (performed in 83.8% of the patients in the usual care group) or medical therapy (no obstruction-relieving intervention). The longest follow-up reported was 5 years.

**Table 1:** Outline of the RCTs included.

Trial	Design and location	Patient surgical risk	TAVI	Comparator	Primary outcome	Follow-up (longest)
PARTNER 1B	Superiority RCT; multi-centres in the US, Canada and Germany	Surgically inoperable (N = 358)	Valve: balloon expanding Edwards SAPIEN heart-valve Route: transfemoral (TF)	Usual care with medical therapy	All-cause mortality over the duration of the trial (endpoints mostly consistent with Valve Academic Research Consortium (VARC) 2 [26, 35])	5 years
PARTNER 1A	Non-inferiority RCT; multi-centres in the US, Canada and Germany	Surgically operable at a high risk (N = 699)	Valve: balloon expanding Edwards SAPIEN heart-valve Route: TF or trasapical (TA)	SAVR	All-cause mortality at 1 year (endpoints mostly consistent with Valve Academic Research Consortium (VARC) 2 [26, 35])	5 years
US CoreValve*	Non- inferiority RCT; multi-centres in the US	Surgically operable at a high risk (N = 795)	Valve: self-expanding Medtronic CoreValve Route: TF or non-TF	SAVR	All-cause mortality at 1 year (endpoints followed VARC 1 [29, 36])	3 years

N = number of patients; RCT = randomised controlled trial; SAVR = surgical aortic valve replacement; TA = transapical; TAVI = transcatheter aortic valve implantation; TF = transfemoral.

* Non-TF included subclavian artery or direct aortic approach.

A total of 1,494 patients surgically operable at a high risk were included in the PARTNER 1A and the US CoreValve trials (Table 1). For the TAVI procedure, a balloon expanding valve and the TF and transapical (TA) routes were used in the PARTNER 1A trial, whist a self-expanding valve and the TF and non-TF route were used in the US CoreValve trial. The longest follow-up was 5 years in the PARTNER 1A trial and 3 years in the US CoreValve trial.

### Assessment of risk of bias in individual studies

The randomisation methods were clearly described but concealment of allocation was not stated in all 3 RCTs. Due to the nature of the interventions, blinding of the patients and personnel would not be feasible. Blinding of the outcome assessment was applied in all 3 trials. Risk of attrition bias was low. Overall, the trials were considered to be of good quality (S3 Table).

### Patient baseline characteristics

The mean age of patients enrolled in the 3 RCTs ranged from 83.1 to 84.5 years, and around half of the patients were female. The majority of patients were in NYHA functional class III or IV (92.8% in PARTNER 1B, 94.2% in PARTNER 1A and 83.8% in US CoreValve). All 3 RCTs included patients with comorbidities including coronary artery disease, atrial fibrillation, kidney disease, chronic obstructive pulmonary disease and previous cardiac surgery such as percutaneous coronary intervention and coronary artery bypass grafting. There was some overlap in the risk stratification profiles between RCTs in inoperable and high surgical risk but operable patients (S4 Table). Only the PARTNER 1A trial evaluated patient baseline characteristics according to access site for TAVI. The TF route was used in more patients (n = 492) than the TA route (n = 207). Differences in baseline characteristics were observed between patients having TAVI via the TF or TA route for age (TF versus TA: 84.4 ± 6.7 years versus 83.2 ± 6.5 years, p = .03), prior coronary bypass grafting (CABG) (39.4% versus 52.9%, p < .001), cerebral vascular disease (25.4% versus 35.7%, p = 0.01), peripheral vascular disease (34.9% versus 60.2%, p < .001), and mean aortic valve gradient (mm Hg) (44.0 ± 14.7 versus 41.0 ± 13.7, p = 0.02).

### Main outcomes in surgically inoperable patients: Mortality

There was no statistically significant difference in 30-day mortality between the TAVI and medical therapy (TAVI versus medical therapy: 2.6% versus 5.9%, p = .09). Compared with medical therapy, TAVI was associated with a statistically significantly lower hazard of death of both all-cause and cardiac-cause at follow-up of 1, 2, 3 and 5 years (Table 2). More details are provided in the S5 Table.

**Table 2:** Mortality: TAVI versus medical therapy in surgically inoperable patients.

	All-cause mortality		Cardiac related mortality	
Follow-up	Hazard ratio	[95% CI]	Hazard ratio	(95% CI)
1 year	0.58	[0.36 to 0.92] p = 0.02	0.39	[0.27 to 0.56]p < .001
2 year	0.50	[0.39 to 0.65] p < .001	0.44	[0.32 to 0.60] p < .001
3 years	0.53	[0.41 to 0.68] P < .001	0.41	[0.30 to 0.56] P < .001
5 years	0.50	[0.39 to 0.65] p < .001	0.41	[0.31 to 0.55]P < .001

All-cause mortality outcome was graded as moderate quality using the GRADE evaluation (S6 Table).

### Secondary outcomes in surgically inoperable patients

#### NYHA classification

Compared with the medical therapy group, the TAVI group had a statistically significantly lower proportion of patients in NYHA classes III/IV at 1, 2 and 3 years and higher proportion of patients in NYHA classes I and II at 5 years of follow-up (S7 Table).

#### Quality of life

TAVI was superior to medical therapy in QoL at least for 1 year, with the Kansas City Cardiomyopathy Questionnaire (KCCQ) summary score being 26 points higher, the 12-Item Short Form Health Survey (SF-12) physical score 5.7 points higher and SF-12 mental health 6.4 points higher in the TAVI group than in the medical therapy group at 1 year (p < .001 for all the three QoL comparisons) (S8 Table).

#### Stroke

TAVI was associated with statistically significantly higher hazard of stroke at 1 year (11.2% versus 5.5%, p *=* .06), 2 years (HR, 2.79; 95% CI, 1.25 to 6.22, p = .009) and 3 years (HR, 2.81; 95% CI, 1.26 to 6.26, p = .012) of follow-up, with the difference becoming non-significant at 5 years.

#### Other secondary outcomes

TAVI was associated with a statistically significantly higher risk of major bleeding up to 1 year of follow-up (24.2% versus 14.9%, p *=* .04), with the difference becoming non-significant between the treatment groups at 2 years and 3 years (cumulative incidence 32.0% for TAVI and 32.9% for SAVR, p *=* .92). The hazard of major vascular complications, reported for 3 years of follow-up only, was significantly higher in the TAVI group than in the medical therapy group (HR, 8.27; 95% CI, 2.92 to 23.44, p = .001). Patients in the TAVI group had a lower hazard of re-hospitalisation due to aortic stenosis or TAVI complication at 1 year (27.0% versus 53.9%, p < .001), 2 years (HR, 0.41; 95% CI, 0.30 to 0.58, p < .001), 3 years (43.5% versus 75.5%, p < .0001) and 5 years (47.6% versus 87.3%, p < .0001) of follow-up. There were no statistically significant differences between the two treatment groups in the risk of permanent pacemaker implantation, myocardial infarction, acute kidney injury or endocarditis at 1, 2 and 3 years (S9–S12 Tables). The included studies did not report the technical success of valve implementation.

The quality of the above secondary outcome measures were graded as moderate (S6 Table).

### Main outcomes in patients surgically operable at a high risk: Mortality

**Fig 2:**
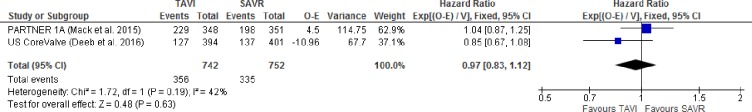
Hazard ratio of all-cause mortality up to 5 years (in patients surgically operable at a high risk).

Time-to-event analysis indicated no statistically significant differences in all-cause mortality between TAVI and SAVR up to 5 years of follow-up (Fig 2). There were no statistically significant differences in either individual or pooled cardiovascular mortality at 1, 2 and 5 years by ITT analysis, although it tended to favour SAVR at 5 years (S1 Fig).

TAVI performed either via the TF or the TA route, showed no statistically significant difference from SAVR in all-cause mortality at follow-ups of 1, 2, and 5 years, and in cardiovascular mortality at 1 and 2 years (S13 Table).

The measure of all-cause mortality was graded as low quality with some uncertainties (S14 Table).

### Secondary outcomes in patients surgically operable at a high risk

#### NYHA classification

Patients who underwent TAVI had a statistically significantly better NYHA classification profile up to 6 months, but thereafter NYHA profiles up to 5 years were similar between the treatment groups (S15 Table).

#### Quality of life

Comparing TAVI using the TF route with SAVR, both the PARTNER 1A and the US CoreValve trials reported a greater improvement on both physical and mental scores SF-12 scores in the TAVI group than in the SAVR group at 1 month follow-up. At 6 months, the only statistically significant difference was reported in the US CoreValve trial for the mental score improvement in the TAVI group compared with the SAVR group. There were no statistically significant differences between TAVI using either the TF or non-TF route and SAVR at 1 year on both physical and mental scores (S16 Table).

TAVI via the TF route was associated with a statistically significant improvement in QoL as measured by EuroQol five-dimension questionnaire (EQ-5D) and Kansas City Cardiomyopathy Questionnaire (KCCQ) at 30 days, which were no longer significant at 6 months or 1 year. There were no statistically significant differences between non-TF TAVI and SAVR in QoL at any of the follow-up points (S2–S5 Figs).

#### Stroke

There were no statistically significant differences between the two treatments in risk of stroke (S6 and S7 Figs).

### Other secondary outcomes

There were no statistically significant differences between the treatments in the risks of major vascular complications and myocardial infarction at all follow-up points (S8 and S9 Figs).

TAVI was associated with better outcomes than SAVR in the overall incidence and severity of prosthesis-patient mismatch up to 2 years of follow-up (S17 Table), but a higher risk of moderate or severe total aortic regurgitation at all follow-up time points up to 3 years (S10 Fig).

Discrepant results were observed in the incidence of major bleeding, variably defined in the included trials, which favoured the TAVI group at all the follow-up time points up to 5 years in the PARTNER 1A trial, but showed no statistically significant differences between the treatments at all the follow-up points up to 3 years in the US CoreValve trial (S11 Fig).

TAVI with a self-expanding valve was associated with a higher incidence of new pacemaker implantation up to 3 years (RR, 3.09; 95% CI, 2.01 to 4.76; RR, 2.28; 95% CI, 1.59 to 3.25; RR, 2.33; 95% CI, 1.66 to 3.25; RR, 2.26; 95% CI, 1.64 to 3.11 at 30 days and 1, 2 and 3 years respectively), but lower incidence of acute kidney injury up to 3 years (RR, 0.43; 95% CI, 0.27 to 0.69; RR, 0.43; 95% CI, 0.27 to 0.69; RR, 0.45; 95% CI, 0.29 to 0.72; RR, 0.45; 95% CI, 0.29 to 0.72 at 30 days and 1, 2 and 3 years respectively). Whereas TAVI with a balloon-expanding valve had no statistically significant differences from SAVR in terms of new pacemaker implantation (S12 Fig) and acute kidney injury at all the follow-up points up to 5 years.

GRADE evaluation showed the above secondary outcome measures were of very low to moderate quality (S14 Table).

## Discussion

We systematically reviewed the evidence on the efficacy and safety of TAVI for aortic stenosis and report here results for patients considered surgically inoperable and patients surgically operable but at a high risk. Without intervention addressing outflow obstruction, the prognosis for these patients is extremely poor but evidence for patients falling in these surgical risk groups has not previously been reviewed. Our analyses based on surgical risk stratification found that all-cause mortality up to 5 years of follow-up did not differ significantly between TAVI and SAVR in patients surgically operable at a high risk, but favoured TAVI over medical therapy in patients surgically inoperable. Although TAVI was non-inferior to SAVR in patients surgically operable at a high risk, shorter term benefits were observed for those patients undergoing TAVI regarding NYHA classification, QoL, overall incidence and severity of prosthesis-patient mismatch and lower incidence of acute kidney injury.

Our systematic review was conducted meticulously using appropriate methodology and the search was comprehensive. A more comprehensive set of efficacy and safety outcomes than those in previous systematic reviews on this topic [4,5] were included. This provides more balanced information on the benefits and harms of TAVI compared with medical therapy or SAVR for aortic stenosis. Furthermore, we compiled all available outcome data up to 5 years of follow-up. There were however insufficient studies for formal assessment of publication bias. Matched observational studies, although were sought to address our specific review questions, were excluded due to unclear risk stratification. There was also some overlap in surgical risk categories across the RCTs in our review. This resulted from variations in trial inclusion criteria but also from a lack of precision in current risk stratification tools for patients with symptomatic aortic valve disease. Given this imprecision, it is difficult to delineate risk groups clearly in study level systematic reviews and meta-analyses. Thus, there is some overlap between risk stratification groups. An individual patient data meta-analysis with sufficiently wide inclusion criteria could provide more definitive indications on the safety and efficacy of TAVI for different surgical risk groups and assist in an improved patient stratification for these patient populations.

Gargiulo and colleagues recently conducted a systematic review of evidence on the safety and efficacy of TAVI compared with SAVR for patients surgically for all patients.[4] Based on RCT data from a total of 4,822 patients, they found no statistically significant differences between TAVI and SAVR in either midterm (> 30 day and ≤ 1 year) or long-term follow-up (> 1 year and up to 5 years). Although they carried out subgroup analysis for low-to-intermediate risk patients they did not do so for high risk subgroups. Our analysis focusing specifically on surgically operable patients at high-risk has particular clinical importance as the available trials were designed to evaluate TAVI as non-inferior to SAVR.

Information comparing TAVI with SAVR using different TAVI routes, valves and delivery sheathes was limited. Our review aimed to evaluate the relative efficacy and safety of TAVI when compared to medical therapy or SAVR and not studies comparing different TAVI procedures. Evidence on which TAVI route, valve and delivery sheath may be superior could be derived from direct comparisons between TAVI procedures performed using such devices. There were also limited data by patient characteristics such as left ventricular ejection fraction, previous coronary artery bypass grafting, diabetes, prosthesis-patient mismatch and sex. Ancillary variations of the TAVI procedure (such as types of anaesthetic, types of imaging examination or guidance, and learning curve) may influence the outcomes compared to the results of the studies we have included in our analyses. For example, there is a growing experience in TAVI via the TF route using local anaesthesia/sedation rather than general anaesthesia, and this may be associated with more rapid recovery and shorter length of stay than general anaesthesia.[37, 38]

From the RCT data available, we have been able to analyse a range of outcomes up to 5 year follow-up, hence there is some uncertainty concerning longer term outcomes of TAVI. Given TAVI candidates have a poor prognosis and the RCTs’ populations had a high mean age, the relevance of results beyond this time point may be dependent on competing risks, particularly in patients considered inoperable. While there was some RCT evidence on TAVI using the TF route and less on the TA route, greater precision on outcomes using specific routes in different risk populations would be desirable. Likewise, greater precision in the quantification of some safety outcomes would facilitate the characterisation of the risk and benefit profiles of SAVR and TAVI.

In conclusion, evidence to date supports the use of TAVI in patients who are surgically inoperable. In patients suitable for surgery but at a high risk, there were short-term advantages in the efficacy of TAVI over SAVR and mixed evidence on safety outcomes.

## Supporting information

S1 Fig Cardiovascular mortality: TAVI versus SAVR (operable at a high risk). (DOCX)

S2 Fig Mean change of EQ-5D from baseline: TF TAVI versus SAVR (operable at a high risk). (DOCX)

S3 Fig Mean change of EQ-5D from baseline: Non-TF TAVI versus SAVR (operable at a high risk). (DOCX)

S4 Fig KCCQ score: TF TAVI versus SAVR (operable at a high risk). (DOCX)

S5 Fig KCCQ score: Non-TF TAVI versus SAVR (operable at a high risk). (DOCX)

S6 Fig All stroke: TAVI versus SAVR (operable at a high risk). (DOCX)

S7 Fig Major stroke: TAVI versus SAVR (operable at a high risk). (DOCX)

S8 Fig Major vascular complications: TAVI versus SAVR (operable at a high risk). (DOCX)

S9 Fig Myocardial infarction: TAVI versus SAVR (operable at a high risk). (DOCX)

S10 Fig Moderate or severe aortic regurgitation: TAVI versus SAVR (operable at a high risk). (DOCX)

S11 Fig Major bleeding: TAVI versus SAVR (operable at a high risk). (DOCX)

S12 Fig Permanent pacemaker implantation: TAVI versus SAVR (operable at a high risk). (DOCX)

S1 Table PRISMA 2009 checklist. (DOC)

S2 Table Search strategy used in the current systematic review and meta-analysis. (DOCX)

S3 Table Risk of bias of the RCTs. (DOCX)

S4 Table Baseline patient characteristics of the RCTs. (DOCX)

S5 Table Mortality of TAVI versus medical therapy (surgically inoperable). (DOCX)

S6 Table GRADE for key outcomes: TAVI versus medical therapy for severe aortic stenosis (surgically inoperable). (DOCX)

S7 Table NYHA classification: TAVI versus medical management (surgically inoperable). (DOCX)

S8 Table Quality of life: TAVI versus medical therapy (surgically inoperable). (DOCX)

S9 Table Permanent pacemaker implantation: TAVI versus medical therapy (surgically inoperable). (DOCX)

S10 Table Myocardial infarctions: TAVI versus medical therapy (surgically inoperable). (DOCX)

S11 Table Renal failure: TAVI versus medical therapy (surgically inoperable). (DOCX)

S12 Table Endocarditis: TAVI versus medical therapy (surgically inoperable). (DOCX)

S13 Table Mortality by TAVI vascular access route (operable at a high risk). (DOCX)

S14 Table GRADE for key outcomes: TAVI compared with SAVR for severe aortic stenosis (operable at a high risk). (DOCX)

S15 Table Proportion of patients in NYHA classes: TAVI versus SAVR (operable at a high risk). (DOCX)

S16 Table Mean change in SF-12 from baseline: TAVI versus SAVR (operable at a high risk). (DOCX)

S17 Table Incidence and severity of prosthesis-patient mismatch: TAVI versus SAVR (operable at a high risk). (DOCX)
