# Supplementary figures and images for: Transcatheter aortic valve implantation for aortic stenosis in high surgical risk patients: A systematic review and meta-analysis
Source: PLoS One. 2018 May 10;13(5):e0196877. doi: 10.1371/journal.pone.0196877 (PMC5944928; doi:10.1371/journal.pone.0196877)

**S1 Fig. Cardiovascular mortality: TAVI versus SAVR (operable at a high risk)**


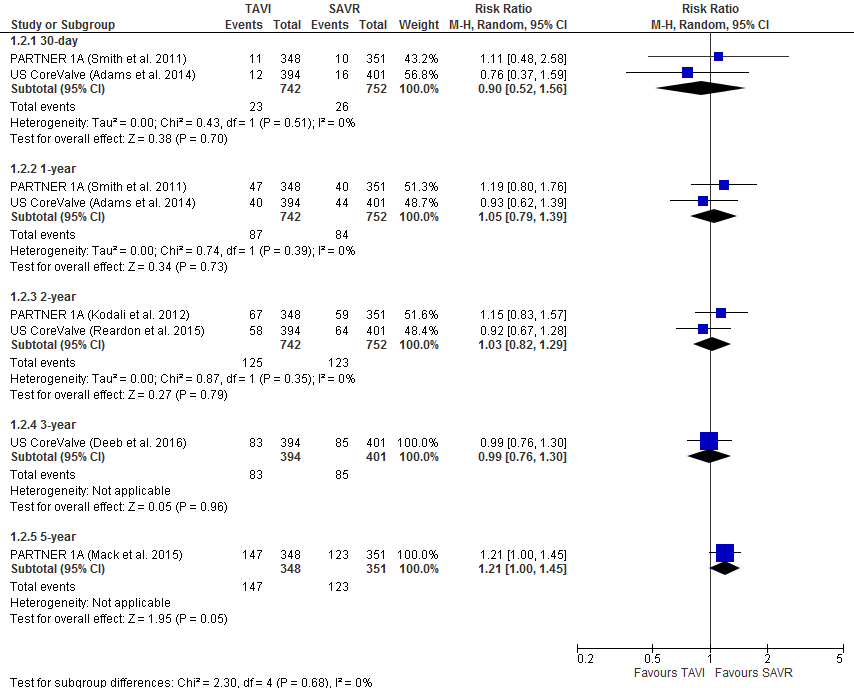

Supplement: S1 Fig — (DOCX) [file pone.0196877.s001.docx]

**S6 Fig. All stroke: TAVI versus SAVR (operable at a high risk)**


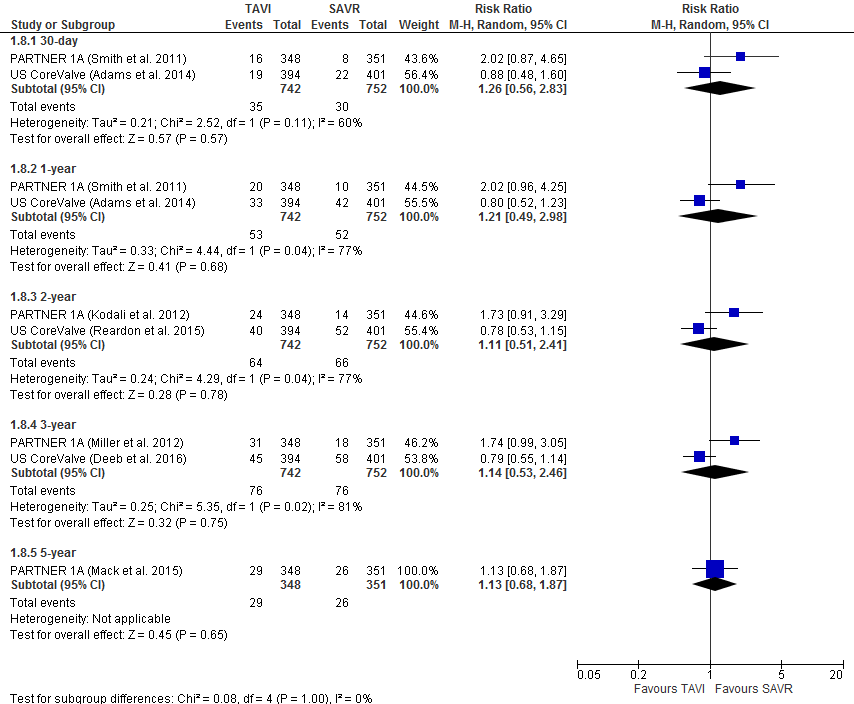

Supplement: S6 Fig — (DOCX) [file pone.0196877.s006.docx]

**S7 Fig. Major stroke: TAVI versus SAVR (operable at a high risk)**


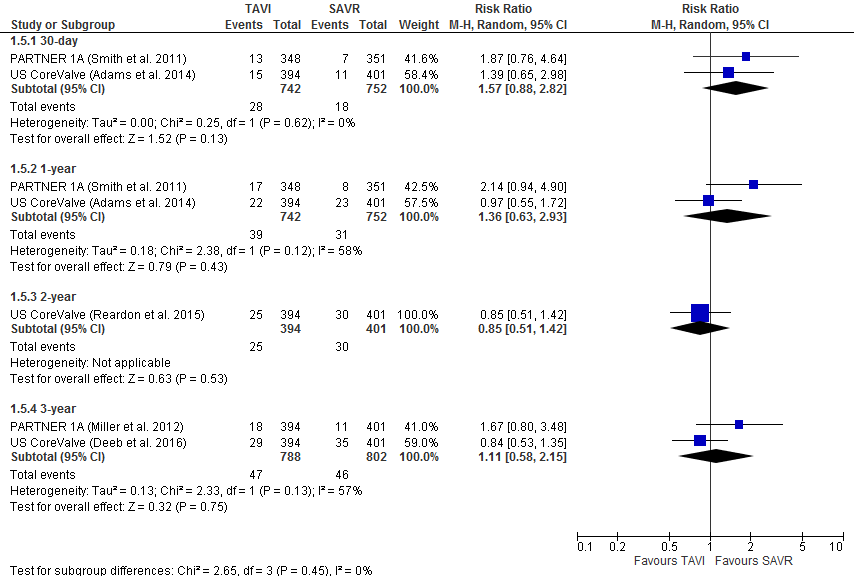

Supplement: S7 Fig — (DOCX) [file pone.0196877.s007.docx]

**S8 Fig. Major vascular complications: TAVI versus SAVR (operable at a high risk)**


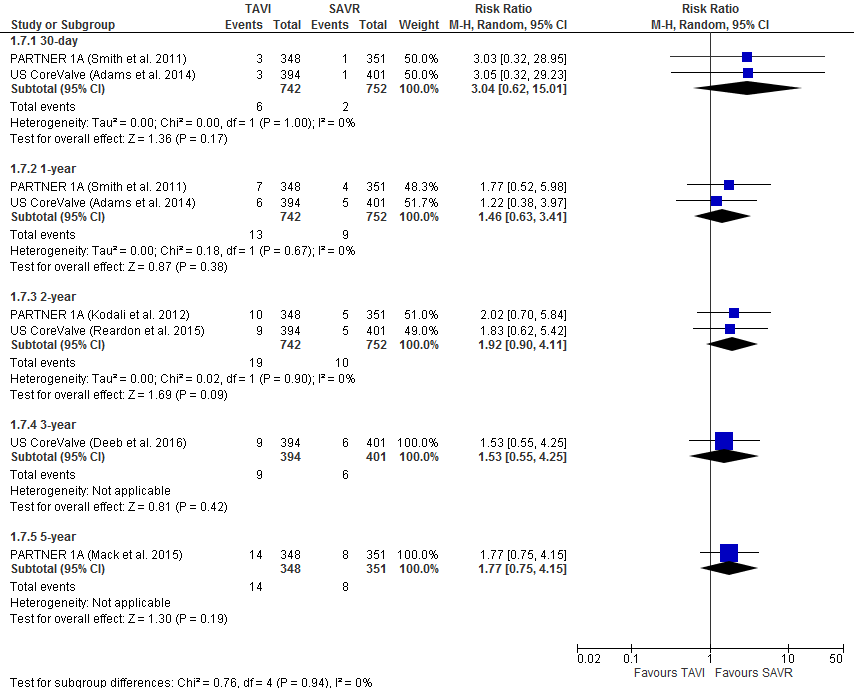

Supplement: S8 Fig — (DOCX) [file pone.0196877.s008.docx]

**S9 Fig. Myocardial infarction: TAVI versus SAVR (operable at a high risk)**

| 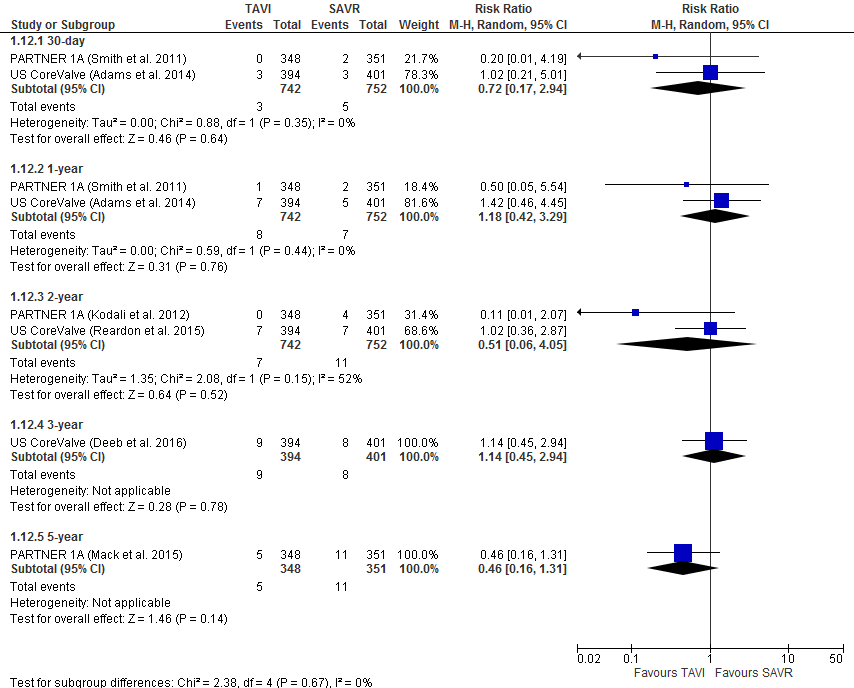 |
| --- |

Supplement: S9 Fig — (DOCX) [file pone.0196877.s009.docx]

**S11 Fig. Major bleeding: TAVI versus SAVR (operable at a high risk)**


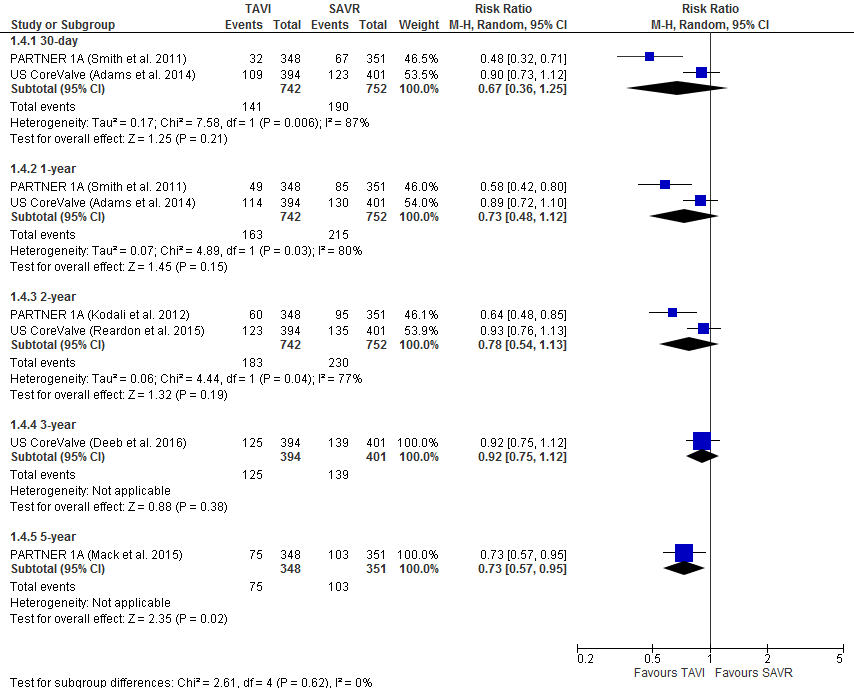

Supplement: S11 Fig — (DOCX) [file pone.0196877.s011.docx]

**S12 Fig. Permanent pacemaker implantation: TAVI versus SAVR (operable at a high risk)**


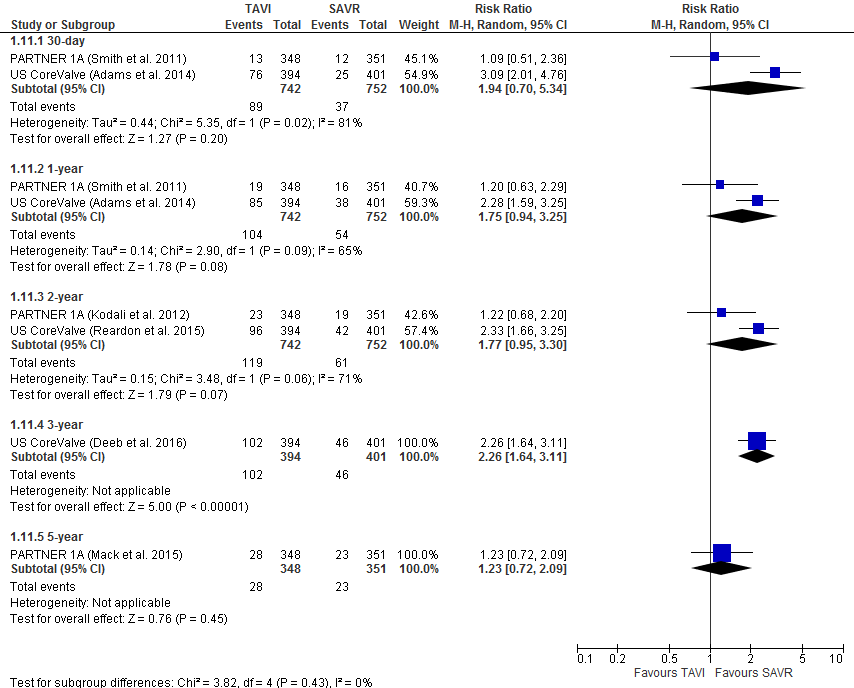

Supplement: S12 Fig — (DOCX) [file pone.0196877.s012.docx]
