# Supplementary material for: Transcatheter aortic valve implantation for aortic stenosis in high surgical risk patients: A systematic review and meta-analysis
Source: PLoS One. 2018 May 10;13(5):e0196877. doi: 10.1371/journal.pone.0196877 (PMC5944928; doi:10.1371/journal.pone.0196877)
Supplement: S2 Fig — (DOCX) [file pone.0196877.s002.docx]

**S2 Fig. Mean change of EQ-5D from baseline: TF TAVI versus SAVR (operable at a high risk)**


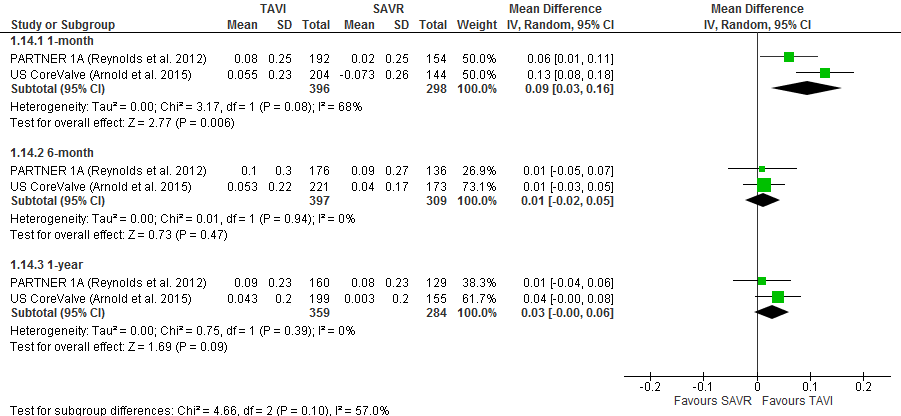


Legend: Standard deviations were calculated for the meta-analysis by the authors of the current review. EQ-5D, EuroQol five dimensions questionnaire. TF, transfemoral.
