# Supplementary material for: Transcatheter aortic valve implantation for aortic stenosis in high surgical risk patients: A systematic review and meta-analysis
Source: PLoS One. 2018 May 10;13(5):e0196877. doi: 10.1371/journal.pone.0196877 (PMC5944928; doi:10.1371/journal.pone.0196877)
Supplement: S3 Fig — (DOCX) [file pone.0196877.s003.docx]

**S3 Fig. Mean change of EQ-5D from baseline: non-TF TAVI versus SAVR (operable at a high risk)**

**
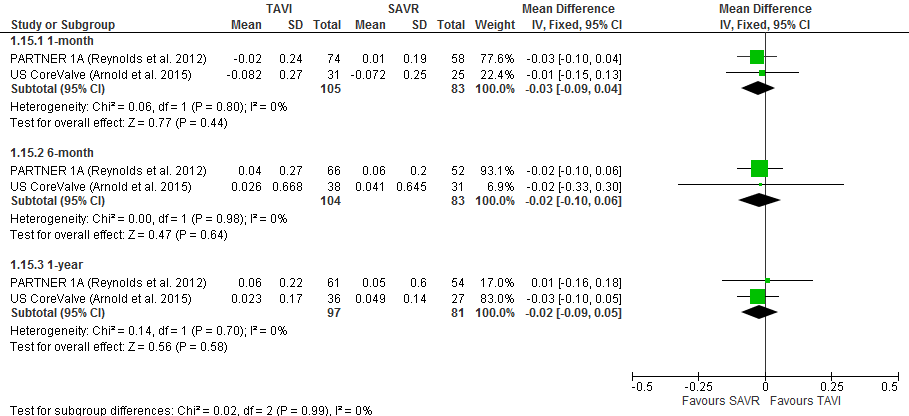
**

Legend: For TAVI the route was transapical in the PARTNER 1A trial and non-transfemoral in the US CoreValve trial. Standard deviations were calculated for the meta-analysis by the authors of the current review. EQ-5D, EuroQol five dimensions questionnaire. Non-TF, non-transfemoral.
