# Supplementary material for: Transcatheter aortic valve implantation for aortic stenosis in high surgical risk patients: A systematic review and meta-analysis
Source: PLoS One. 2018 May 10;13(5):e0196877. doi: 10.1371/journal.pone.0196877 (PMC5944928; doi:10.1371/journal.pone.0196877)
Supplement: S10 Fig — (DOCX) [file pone.0196877.s010.docx]

**S10 Fig. Moderate or severe aortic regurgitation: TAVI versus SAVR (operable at a high risk)**


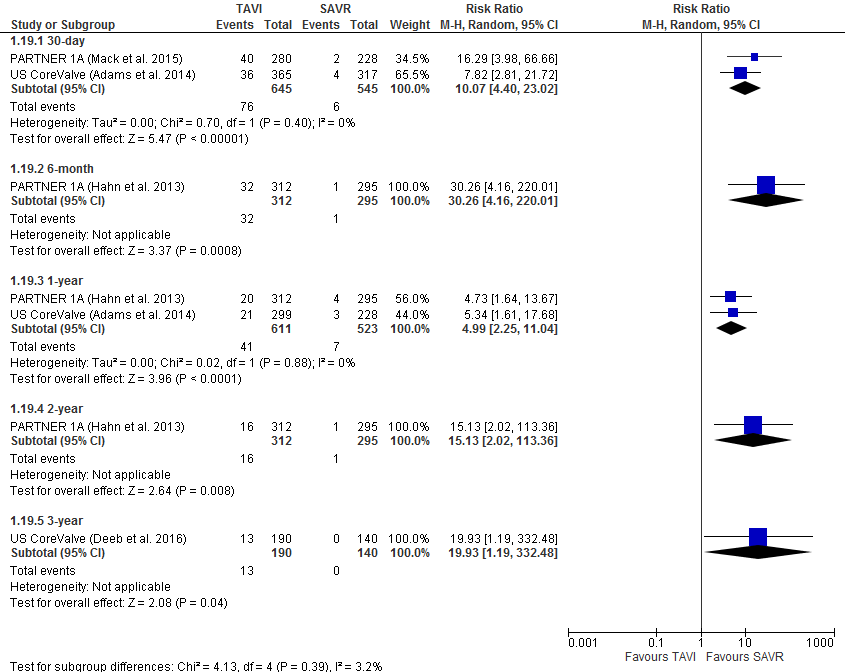


Legend: Based on number of patients in each treatment group who had echocardiography study.
