# Supplementary material for: Transcatheter aortic valve implantation for aortic stenosis in high surgical risk patients: A systematic review and meta-analysis
Source: PLoS One. 2018 May 10;13(5):e0196877. doi: 10.1371/journal.pone.0196877 (PMC5944928; doi:10.1371/journal.pone.0196877)
Supplement: S2 Table — (DOCX) [file pone.0196877.s014.docx]

**S2 Table. Search strategy used in the current systematic review and meta-analysis.**

**Database: MEDLINE (Ovid) 1946 to July Week 3 2016**

1 Aortic valve/ab

2 heart valve diseases/ or exp aortic valve stenosis/

3 (aortic* adj stenosis).tw.

4 (valv* adj3 disease).tw.

5 or/1-4

6 ((percutan* or transcath*) adj3 (heart* or aortic*) adj3 valve*).tw.

7 ((percutan* or transcath*) adj3 valve*).tw.

8 PAVR.tw.

9 TAVR.tw.

10 TAVI.tw.

11 ((transap* or transventric* or percutan* or transcath*) adj3 (deliver* or access* or approach* or minimal*)).tw.

12 or/6-11

13 5 and 12

14 animals/ not humans/

15 13 not 14

16 limit 15 to yr="2002 - current"

**Database: Ovid MEDLINE In-Process & Other Non-Indexed Citations August 01, 2016**

1 (Aortic valve* adj3 abnormal*).tw.

2 (aortic* adj stenosis).tw.

3 (valv* adj3 disease).tw.

4 or/1-3

5 ((percutan* or transcath*) adj3 (heart* or aortic*) adj3 valve*).tw.

6 ((percutan* or transcath*) adj3 valve*).tw.

7 PAVR.tw.

8 TAVR.tw.

9 TAVI.tw)

10 ((transap* or transventric* or percutan* or transcath*) adj3 (deliver* or access* or approach* or minimal*)).tw.

11 or/5-10

12 4 and 11

13 limit 12 to yr="2002 - current"

**Database: Embase (Ovid) 1974 to 2016 August 01**

1 aorta valve/

2 exp valvular heart disease/

3 aorta valve stenosis/

4 (aortic* adj stenosis).mp.

5 (aortic adj stenosis).tw.

6 (valv* adj3 disease).tw.

7 or/1-5

8 ((percutan* or transcath*) adj3 (heart* or aortic*) adj3 valve*).tw.

9 ((percutan* or transcath*) adj3 valve*).tw.

10 PAVR.tw.

11 TAVR.tw.

12 TAVI.tw.

13 ((transap* or transventric* or percutan* or transcath*) adj3 (deliver* or access* or approach* or minimal*)).tw.

14 or/8-13

15 7 and 14

16 animals/ not humans/

17 15 not 16

18 limit 17 to yr="2002 - 2016"

**Database: Cochrane Library (Wiley): CENTRAL Issue 7 of 12 July 2016, CDSR Issue 8 of 12 August 2016, HTA Issue 3 of 4 July 2016, DARE Issue 2 of 4 (April 2015), EED Issue 2 of 4 April 2015**

Searched 8 August 2016

#1 MeSH descriptor: [Aortic Valve] explode all trees and with qualifier(s): [Abnormalities - AB]

#2 MeSH descriptor: [Heart Valve Diseases] this term only

#3 MeSH descriptor: [Aortic Valve Stenosis] explode all trees

#4 aortic* near/3 stenosis

#5 valv* near/3 disease

#6 #1 or #2 or #3 or #4 or #5

#7 (percutan* or transcath*) near/3 (heart* or aortic*) near/3 (valve*)

#8 (percutan* or transcath*) near/3 (valve*)

#9 PAVR or TAVI or TAVR

#10 (transap* or transventric* or percutan* or transcath*) near/3 (deliver* or access* or approach* or minimal*)

#11 #7 or #8 or #9 or #10

#12 #6 and #11

#13 #6 and #11 Publication Year from 2002 to 2016

**Database: PubMed (US NLM)**

Searched 9 August 2016

#1 Search ((aortic valve stenosis[MeSH Terms]) OR "aortic valve/abnormalities"[MeSH Terms]) OR heart valve diseases[MeSH Terms]

#2 Search (((valv* disease* OR aortic stenosis) and publisher [SB]))

#3 Search (#1 or #2)

#4 Search ((percutaneous or transcath*) AND (valve* OR heart OR aortic) *) and publisher[SB])

#5 Search TAVI or TAVR or PAVR) and publisher[SB]

#6 Search (transap* OR transcentric OR percutan* OR transcath*) and publisher[SB] AND (deliver* OR access* OR approach* OR minimal*) and publisher[SB]

#7 Search (#4 or #5 or #6)

#8 Search (#7 and #3)

#9 Search (#7 and #3) Sort by: Author Filters: Publication date from 2002/01/01 to 2016/08/31

**Database: ZETOC British Library**

Searched : 8 Aug 2016

Terms used: "aortic valve*" or "aortic stenosis" or "heart valve*" date: 2002-2016 TAVI or TAVR or PAVR date: 2002-2016
