# Supplementary material for: Transcatheter aortic valve implantation for aortic stenosis in high surgical risk patients: A systematic review and meta-analysis
Source: PLoS One. 2018 May 10;13(5):e0196877. doi: 10.1371/journal.pone.0196877 (PMC5944928; doi:10.1371/journal.pone.0196877)
Supplement: S3 Table — (DOCX) [file pone.0196877.s015.docx]

**S3 Table. Risk of bias of the RCTs**

| **Trial** | **Risk item** | **Authors' judgement** | **Support for judgement** |
| --- | --- | --- | --- |
| PARTNER 1B | Random sequence generation (selection bias) | Low risk | Computer-generated randomized blocks at each site and for each subgroup |
|  | Allocation concealment (selection bias) | Unclear risk | Not specified |
|  | Blinding of participants and personnel (performance bias) | High risk | Not blinded |
|  | Blinding of outcome assessment (detection bias) | Low risk | Independent Data Safety Monitoring Board reviewed all safety data |
|  | Incomplete outcome data (attrition bias) | Low risk |  |
|  | Selective reporting (reporting bias) | Unclear risk | QoL not reported |
|  | Other bias | High risk | Supported by Edwards Lifesciences |
| PARTNER 1A | Random sequence generation (selection bias) | Low risk | Computer-generated randomized blocks at each site and for each subgroup |
|  | Allocation concealment (selection bias) | Unclear risk | Not specified |
|  | Blinding of participants and personnel (performance bias) | High risk | Not blinded |
|  | Blinding of outcome assessment (detection bias) | Low risk | Independent Data Safety Monitoring Board reviewed all safety data |
|  | Incomplete outcome data (attrition bias) | Low risk |  |
|  | Selective reporting (reporting bias) | Low risk | QoL reported in Reynolds 2012 |
|  | Other bias | High risk | Supported by Edwards Lifesciences |
| US CoreValve | Random sequence generation (selection bias) | Low risk | Assigned a patient identification number in the interactive voice/web randomization service (IXRS) |
|  | Allocation concealment (selection bias) | Unclear risk | Not specified |
|  | Blinding of participants and personnel (performance bias) | High risk | Not blinded |
|  | Blinding of outcome assessment (detection bias) | Low risk | Independent Data Safety Monitoring Board performed comprehensive data reviews |
|  | Incomplete outcome data (attrition bias) | Low risk |  |
|  | Selective reporting (reporting bias) | Low risk |  |
|  | Other bias | High risk | Medtronic funded the trial and developed the protocol in collaboration with the study steering committee. Medtronic was responsible for the selection of the clinical sites, monitoring of the data, and management of all source data and statistical analyses |
