# Supplementary material for: Transcatheter aortic valve implantation for aortic stenosis in high surgical risk patients: A systematic review and meta-analysis
Source: PLoS One. 2018 May 10;13(5):e0196877. doi: 10.1371/journal.pone.0196877 (PMC5944928; doi:10.1371/journal.pone.0196877)
Supplement: S4 Table — (DOCX) [file pone.0196877.s016.docx]

**S4 Table. Baseline patient characteristics of the RCTs**

| **Study and comparison** | **Female (%)** | **Age (year), mean (SD)** | **NYHA III/IV (%)** | **Logistic EuroSCORE, % (SD)** | **STS, mean (SD)** | **CAD, (%)** | **AF,**  **(%)** | **Diabetes (%)** | **Kidney disease (%)** | **COPD (%)** | **Previous cardiac surgery (%)** |
| --- | --- | --- | --- | --- | --- | --- | --- | --- | --- | --- | --- |
| PARTNER 1B *(in patients unsuitable for SAVR)* | | | | | | | | | | | |
| - TAVI | 54.2 | 83.1 (8.6) | 92.2 | NR | 11.2 (5.8) | 27.4 | 32.9 | NR | 5.6 | 41.3 | PCI: 30.5; CABG: 37.4 |
| - Medical therapy | 53.1 | 83.2 (8.3) | 93.9 | NR | 12.1 (6.1) | 27.5 | 48.8 | NR | 9.6 | 52.5 | PCI: 24.8; CABG: 45.6 |
| PARTNER 1A *(in patients for whom SAVR is suitable but poses a high risk)* | | | | | | | | | | | |
| - TAVI | 42.2 | 83.6 (6.8) | 94.3 | 29.3 (16.5) | 11.8 (3.3) | 74.9 | 40.8 | NR | 11.1 | 43.4 | PCI: 34.0; CABG: 42.6 |
| - SAVR | 43.3 | 84.5 (6.4) | 94.0 | 29.2 (15.6) | 11.7 (3.5) | 76.9 | 42.7 | NR | 7.0 | 43.0 | PCI: 32.5; CABG: 44.2 |
| US CoreValve *(in patients for whom SAVR is suitable but poses a high risk)* | | | | | | | | | | | |
| - TAVI | 46.4 | 83.2 (7.1) | 80.7 | 17.6 (13.0) | 7.3 (3.0) | 75.4 | 40.0 | 34.9 | 12.3 (stage 4/5) | 13.3 | PCI: 33.8; CABG: 29.7 |
| - SAVR | 47.1 | 83.5 (6.3) | 86.8 | 18.4 (12.8) | 7.5 (3.2) | 76.3 | 47.5 | 45.4 | 13.1 (stage 4/5) | 9 | PCI: 37.9; CABG: 30.2 |
| Legend: AF, atrial fibrillation; CABG, coronary artery bypass grafting; CAD, coronary artery disease; COPD, Chronic obstructive pulmonary disease; EuroSCORE, European System for Cardiac Operative Risk Evaluation; NR, not reported; PCI, percutaneous coronary intervention; STS, Society of Thoracic Surgeons (predictor risk of mortality) ; SAVR, surgical aortic valve replacement; TAVI, transcatheter aortic valve implantation. | | | | | | | | | | | |
