# Supplementary material for: Transcatheter aortic valve implantation for aortic stenosis in high surgical risk patients: A systematic review and meta-analysis
Source: PLoS One. 2018 May 10;13(5):e0196877. doi: 10.1371/journal.pone.0196877 (PMC5944928; doi:10.1371/journal.pone.0196877)
Supplement: S5 Table — (DOCX) [file pone.0196877.s017.docx]

**S5 Table. Mortality of TAVI versus medical therapy (surgically inoperable)**

| **Follow-up** | **Reference paper** | **TAVI (n=179)** | **Medical therapy (n=179)** | **Analysis, HR (95% CI)** |
| --- | --- | --- | --- | --- |
| ***At 1-year:*** | Makkar et al. 2012 |  |  |  |
| - All-cause |  | 30.7% | 50.7% | 0.58 (0.36 to 0.92), *P* = .02 |
| ***At 2-year:*** | Makkar et al. 2012 |  |  |  |
| - All-cause |  | 43.3% | 68.0% | 0.56 (0.43 to 0.73), *P* < .001 |
| - Cardiac cause |  | 31.0% | 62.4% | 0.44 (0.32 to 0.60), *P* < .001 |
| ***At 3-year:*** | Kapadia et al. 2014 |  |  |  |
| - All-cause |  | 54.1% | 80.9% | 0.53 (0.41 to 0.68), *P* < .0001 |
| - Cardiac cause |  | 41.4% | 74.5% | 0.41 (0.30 to 0.56), *P* < .0001 |
| ***At 5-year:*** | Kapadia et al. 2015 |  |  |  |
| - All-cause |  | 71.8% | 93.6% | 0.5 (0.39 to 0.65), *P* < .0001 |
| - Cardiac cause |  | 57.5% | 85.9% | 0.41 (0.31 to 0.55), *P* < .0001 |
| Legend: Percentages shown are Kaplan–Meier estimates. CI, confidence interval; HR, hazard ratio; TAVI, transcatheter aortic valve implantation. | | | | |
