# Supplementary material for: Transcatheter aortic valve implantation for aortic stenosis in high surgical risk patients: A systematic review and meta-analysis
Source: PLoS One. 2018 May 10;13(5):e0196877. doi: 10.1371/journal.pone.0196877 (PMC5944928; doi:10.1371/journal.pone.0196877)
Supplement: S6 Table — (DOCX) [file pone.0196877.s018.docx]

**S6 Table. GRADE for key outcomes: TAVI versus medical therapy for severe aortic stenosis (surgically inoperable)**

| **Quality assessment** | | | | | | | **Impact** | **Quality** | **Importance** |
| --- | --- | --- | --- | --- | --- | --- | --- | --- | --- |
| **№ of studies** | **Study design** | **Risk of bias** | **Inconsistency** | **Indirectness** | **Imprecision** | **Other considerations** |  |  |  |
| ***All-cause mortality (follow up: 5 years)*** | | | | | | | | | |
| 1 | randomised trials | serious ^a,b,c^ | not serious | not serious | not serious | none | TAVI (71.8%) versus medical therapy (93.6%); HR 0.50, 95% CI 0·39 to 0·65; *P* < .0001 | ⨁⨁⨁◯ MODERATE | CRITICAL |
| ***Quality of life (not reported)*** | | | | | | | | | |
| 1 | randomised trials | serious ^a,b,c^ | not serious | not serious | not serious | none | KCCQ summary score was 26 points higher, SF-12 physical score 5.7 points higher and SF-12 mental health 6.4 points higher with than with the control at 1 year (*P* < .001 for all the three comparisons). | ⨁⨁⨁◯ MODERATE | IMPORTANT |
| ***30-day mortality (follow up: 30 days)*** | | | | | | | | | |
| 1 | randomised trials | serious ^a,b,c^ | not serious | not serious | not serious | none | TAVI versus medical therapy: 2.6% versus 5.9%, *P* = .09 | ⨁⨁⨁◯ MODERATE | CRITICAL |
| ***Major bleeding (follow up: 3 years)*** | | | | | | | | | |
| 1 | randomised trials | serious ^a,b,c^ | not serious | not serious | not serious | none | TAVI (32.0%) versus medical therapy (32.9%); HR, 1.69; 95% CI 1.06 to 2.70; *P* = .03 | ⨁⨁⨁◯ MODERATE | IMPORTANT |
| ***Stroke (follow up: 5 years)*** | | | | | | | | | |
| 1 | randomised trials | serious ^a,b,c^ | not serious | not serious | not serious | none | The hazard of stroke higher was higher with TAVI than medical therapy up to 3 years (HR 2.81, 95% CI 1.26 to 6.26, *P* = .012), then the significance did not maintain at 5 years. | ⨁⨁⨁◯ MODERATE | IMPORTANT |
| ***New permanent pacemaker implantation (follow up: 3 years)*** | | | | | | | | | |
| 1 | randomised trials | serious ^a,b,c^ | not serious | not serious | not serious | none | No statistically significant differences between TAVI and SAVR in the proportion of patients requiring PPI at 1 year (4.7% vs 8.6%), 2 years (6.4% vs 8.6%) and 3 years (7.6% vs 8.6%) | ⨁⨁⨁◯ MODERATE | IMPORTANT |
| ***Repeat hospital admission (follow up: 5 years)*** | | | | | | | | | |
| 1 | randomised trials | serious ^a,b,c^ | not serious | not serious | not serious | none | TAVI (47.6%) versus medical therapy (87.3%); HR, 0.40; 95% CI, 0.29 to 0.55; *P* < .0001 | ⨁⨁⨁◯ MODERATE | IMPORTANT |

Legend: a, unblended; b, not free from industry funding; c. Allocation concealment process not specified. CI, confidence interval; HR, hazard ratio; TAVI, transcatheter aortic valve implantation; vs, versus.
