# Supplementary material for: Transcatheter aortic valve implantation for aortic stenosis in high surgical risk patients: A systematic review and meta-analysis
Source: PLoS One. 2018 May 10;13(5):e0196877. doi: 10.1371/journal.pone.0196877 (PMC5944928; doi:10.1371/journal.pone.0196877)
Supplement: S7 Table — (DOCX) [file pone.0196877.s019.docx]

**S7 Table. NYHA classification: TAVI versus medical management (surgically inoperable)**

| **Follow-up** | **Reference** | **TAVI**  **n/N (%)** | **Medical therapy**  **n/N (%)** | **Analysis** |
| --- | --- | --- | --- | --- |
| 1-year (NYHA class III/IV) | Makkar et al. 2012 | 23.7% (28/118) | 60.8% (48/79) | *P* < .001 |
| 2-year (NYHA class III/IV) | Makkar et al. 2012 | 16.8% (16/95) | 57.5% (23/40) | *P* < .001 |
| 3-year (NHYA class III/IV) | Kapadia et al. 2014 | 70.3%* | 95.2%* | *P* < .001 |
| 5-year (NHYA class I/II) | Kapadia et al. 2015 | 86% (42/49 survivors) | 60% (3/5 survivors) | *P* < .0001 |
| Legend: * ITT analysis based on 179 patients randomised in each group. N, number of patients; NYHA, New York Heart Association (class) ; TAVI, transcatheter aortic valve implantation. | | | | |
