# Supplementary material for: Transcatheter aortic valve implantation for aortic stenosis in high surgical risk patients: A systematic review and meta-analysis
Source: PLoS One. 2018 May 10;13(5):e0196877. doi: 10.1371/journal.pone.0196877 (PMC5944928; doi:10.1371/journal.pone.0196877)
Supplement: S8 Table — (DOCX) [file pone.0196877.s020.docx]

**S8 Table. Quality of life: TAVI versus medical therapy (surgically inoperable)**

| **Scale** | **Mean difference:**  **TAVI (n=179) - SAVR (n=179)** | **Analysis** |
| --- | --- | --- |
| KCCQ summary |  |  |
| - 1-month | 13.3 | 95% CI 7.6 to 19.0, *P* < .001 |
| - 6-month | 20.8 | 95% CI 14.7 to 27.0, *P* < .001 |
| - 12-month | 26.0 | 95% CI 18.7 to 33.3, *P* < .001 |
| KCCQ quality of life |  |  |
| - 1-month | 14.5 | 95% CI 8.6 to 21.0, *P* < .001 |
| - 6-month | 24.2 | 95% CI 17.4 to 31.0, *P* < .001 |
| - 12-month | 30.5 | 95% CI 22.3 to 38.7, *P* < .001 |
| SF-12 physical |  |  |
| - 1-month | 4.5 | 95% CI 2.5 to 6.6, *P* < .001 |
| - 6-month | 5.5 | 95% CI 3.0 to 7.9, *P* < .001 |
| - 12-month | 5.7 | 95% CI 2.8 to 8.5, *P* < .001 |
| SF-12 mental |  |  |
| - 1-month | 0.6 | 95% -1.6 to 2.6, *P* = .61 |
| - 6-month | 3.2 | 95% CI 1.1 to 5.3, *P* = .003 |
| - 12-month | 6.4 | 95% CI 3.5 to 9.4, *P* < .001 |
| Legend: Positive values indicate better health status with TAVI. CI, confidence interval; KCCQ, Kansas City Cardiomyopathy Questionnaire; SF-12, Short Form-12 General Health Survey; TAVI, transcatheter aortic valve implantation. | | |
