# Supplementary material for: Transcatheter aortic valve implantation for aortic stenosis in high surgical risk patients: A systematic review and meta-analysis
Source: PLoS One. 2018 May 10;13(5):e0196877. doi: 10.1371/journal.pone.0196877 (PMC5944928; doi:10.1371/journal.pone.0196877)
Supplement: S10 Table — (DOCX) [file pone.0196877.s022.docx]

**S10 Table. Myocardial infarctions: TAVI versus medical therapy (surgically inoperable)**

| **Follow-up** | **Reference** | **TAVI (n=179)** | **Medical therapy (n=179)** | **Analysis** |
| --- | --- | --- | --- | --- |
| 1-year | PARTNER 1B (Makkar et al. 2012) | 0.8% | 0.7% | *P* = .91 |
| 2-year | PARTNER 1B (Makkar et al. 2012) | 1.6% | 2.5% | *P* = .69 |
| 3-year | PARTNER 1B (Kapadia et al. 2014) | 4.1% | 2.5% | *P* = .59 |
| Legend: Percentages shown are Kaplan-Meier estimates. N, number of patients; TAVI, transcatheter aortic valve implantation. | | | | |
