# Supplementary material for: Transcatheter aortic valve implantation for aortic stenosis in high surgical risk patients: A systematic review and meta-analysis
Source: PLoS One. 2018 May 10;13(5):e0196877. doi: 10.1371/journal.pone.0196877 (PMC5944928; doi:10.1371/journal.pone.0196877)
Supplement: S13 Table — (DOCX) [file pone.0196877.s025.docx]

**S13 Table. Mortality by TAVI vascular access route (operable at a high risk)**

| **Follow-up** | **Reference for PARTNER 1A** | **TF-TAVI (n=244)** | **SAVR (n=248)** | **Analysis, HR (95% CI)** |
| --- | --- | --- | --- | --- |
| All-cause |  |  |  |  |
| - 1-year | Smith et al. 2011; Kodali et al. 2012 | 22.2% | 26.4% | *P* = .29 |
| - 2-year | Kodali et al. 2012 | 30.9% | 34.6% | *P* = .38 |
| - 5-year | Mack et al. 2015 | 64.38% | 70.21% | 0.91 (0.72 to 1.14), *P* = .41 |
| Cardiovascular cause |  |  |  |  |
| - 1-year | Smith et al. 2011; Kodali et al. 2012 | 12.6% | 13.3% | *P* = .83 |
| - 2-year | Kodali et al. 2012 | 19.5% | 20.6% | *P* = .60 |
| **Follow-up** | **Reference** | **TA-TAVI (n=104)** | **SAVR (n=103)** | **Analysis** |
| All-cause |  |  |  |  |
| - 1-year | Smith et al. 2011; Kodali et al. 2012 | 29.0% | 27.9% | *P* = .85 |
| - 2-year | Kodali et al. 2012 | 41.1% | 35.7% | *P* = .44 |
| - 5-year | Mack et al. 2015 | 85.73% | 67.46% | 1.37 (0.98 to 1.92), *P* = .07 |
| Cardiovascular cause |  |  |  |  |
| - 1-year | Smith et al. 2011; Kodali et al. 2012 | 18.5% | 12.3% | *P* = .24 |
| - 2-year | Kodali et al. 2012 | 26.0% | 20.5% | *P* = .40 |
| Legend: All based on intention-to-treat analysis. Percentages are Kaplan-Meier estimates at the specific time point; p-values are for between-group comparisons of the frequency of the event at each time point (except for the 5-year follow-ups). CI, confidence interval; HR, hazard ratio; n, number of patient; TA-TAVI, transapcial transcatheter aortic valve implantation; TF-TAVI, transfemoral aortic valve implantation; SAVR, surgical aortic valve replacement. | | | | |
