# Supplementary material for: Transcatheter aortic valve implantation for aortic stenosis in high surgical risk patients: A systematic review and meta-analysis
Source: PLoS One. 2018 May 10;13(5):e0196877. doi: 10.1371/journal.pone.0196877 (PMC5944928; doi:10.1371/journal.pone.0196877)
Supplement: S14 Table — (DOCX) [file pone.0196877.s026.docx]

**S14 Table. GRADE for key outcomes: TAVI compared with SAVR for severe aortic stenosis (operable at a high risk)**

| **Quality assessment** | | | | | | | | | | | | | | **№ of patients** | | **Effect** | | | **Quality** | **Importance** |
| --- | --- | --- | --- | --- | --- | --- | --- | --- | --- | --- | --- | --- | --- | --- | --- | --- | --- | --- | --- | --- |
| **№ of studies** | **Study design** | | **Risk of bias** | | **Inconsistency** | | | **Indirectness** | | | **Imprecision** | | **Other considerations** | **TAVI** | **SAVR** | **Relative (95% CI)** | **Absolute (95% CI)** | |  |  |
| ***All-cause mortality (follow up: range 3 years to 5 years)*** | | | | | | | | | | | | | | | | | | | | |
| 2 | randomised trials | | serious ^a,b,c^ | | not serious | | | not serious | | | | serious ^d^ | none | 356/742 (48.0%) | 335/752 (44.5%) | HR 0.97 (0.83 to 1.12) | 10 fewer per 1,000 (from 38 more to 58 fewer) | | ⨁⨁◯◯ LOW | CRITICAL |
| ***Quality of life: EQ-5D (follow-up: 1 year)*** | | | | | | | | | | | | | | | | | | | | |
| 2 | randomised trials | serious ^a,b,c^ | | | not serious | | | not serious | | | | not serious | none | 359 | 284 | - | MD 0.03 more (0 to 0.06 more) | | ⨁⨁⨁◯ MODERATE | IMPORTANT |
| ***30-day mortality (follow up: 30 days)*** | | | | | | | | | | | | | | | | | | | | |
| 2 | randomised trials | serious ^a,b,c^ | | | not serious | | | not serious | | | | not serious | none | 27/742 (3.6%) | 43/752 (5.7%) | RR 0.64 (0.40 to 1.02) | 21 fewer per 1,000 (from 1 more to 34 fewer) | | ⨁⨁⨁◯ MODERATE | CRITICAL |
| ***All stroke (follow up: 2 years)*** | | | | | | | | | | | | | | | | | | | | |
| 2 | randomised trials | serious ^a,b,c^ | | | serious ^e^ | | | not serious | | | | very serious ^d^ | none | 64/742 (8.6%) | 66/752 (8.8%) | RR 1.11 (0.51 to 2.41) | 10 more per 1,000 (from 43 fewer to 124 more) | | ⨁◯◯◯ VERY LOW | IMPORTANT |
| ***Major bleeding (follow up: 2 years)*** | | | | | | | | | | | | | | | | | | | | |
| 2 | randomised trials | | | serious ^a,b,c^ | not serious | not serious | | | not serious | | | | none | 183/742 (24.7%) | 230/752 (30.6%) | RR 0.78 (0.54 to 1.13) | 67 fewer per 1,000 (from 40 more to 141 fewer) | | ⨁⨁⨁◯ MODERATE | IMPORTANT |
| ***Myocardial infarction (follow up: 2 years)*** | | | | | | | | | | | | | | | | | | | | |
| 2 | randomised trials | | | serious ^a,b,c^ | not serious | not serious | | | very serious ^d^ | | | | none | 7/742 (0.9%) | 11/752 (1.5%) | RR 0.51 (0.06 to 4.05) | | 7 fewer per 1,000 (from 14 fewer to 45 more) | ⨁◯◯◯ VERY LOW | IMPORTANT |
| ***Acute kidney injury (follow up: 2 years)*** | | | | | | | | | | | | | | | | | | | | |
| 2 | randomised trials | | | serious ^a,b,c^ | not serious | | not serious | | | serious ^d^ | | | none | 44/742 (5.9%) | 75/752 (10.0%) | RR 0.64 (0.31 to 1.34) | 36 fewer per 1,000 (from 34 more to 69 fewer) | | ⨁⨁◯◯ LOW | IMPORTANT |

Legend: a,unblinded; b, not free from industry funding; c, allocation concealment process not specified; d, a 25% relative risk reduction or increase (RR 0.75 and 1.25); e, statistical heterogeneity between RCTs; CI, confidence interval; HR, hazard ratio; RR, risk ratio; MD, mean difference.
