# Supplementary material for: Transcatheter aortic valve implantation for aortic stenosis in high surgical risk patients: A systematic review and meta-analysis
Source: PLoS One. 2018 May 10;13(5):e0196877. doi: 10.1371/journal.pone.0196877 (PMC5944928; doi:10.1371/journal.pone.0196877)
Supplement: S15 Table — (DOCX) [file pone.0196877.s027.docx]

**S15 Table. Proportion of patients in NYHA classes: TAVI versus SAVR (operable at a high risk)**

| **Follow-up** | **Trial** | **TAVI** | | **SAVR** | | **Analysis** |
| --- | --- | --- | --- | --- | --- | --- |
|  |  | **n. analysed** | **n (%)** | **n. analysed** | **n (%)** |  |
| 1-month |  |  |  |  |  |  |
| - NYHA class I | US CoreValve (Adams el al. 2014) | 337 | 13.4% | 331 | 32.6% | P < .01* |
| - NYHA class II | US CoreValve (Adams el al. 2014) | 376 | 39.4% | 331 | 40.8% |  |
| - NYHA class III | US CoreValve (Adams el al. 2014) | 376 | 13.0% | 331 | 18.4% |  |
| - NYHA class IV | US CoreValve (Adams el al. 2014) | 376 | 0.3% | 331 | 4.2% |  |
| 6-month |  |  |  |  |  |  |
| - NYHA class I | US CoreValve (Adams el al. 2014) | 363 | 5.23% | 315 | 42.5% | *P* = .04* |
| - NYHA class II | US CoreValve (Adams el al. 2014) | 363 | 31.4% | 315 | 33.7% |  |
| - NYHA class III | US CoreValve (Adams el al. 2014) | 363 | 6.1% | 315 | 4.4% |  |
| - NYHA class IV | US CoreValve (Adams el al. 2014) | 363 | 0.3% | 315 | 0.6% |  |
| 12-month |  |  |  |  |  |  |
| - NYHA class I | US CoreValve (Adams el al. 2014) | 365 | 48.2% | 304 | 44.1% | *P* = .10 * |
| - NYHA class II | US CoreValve (Adams el al. 2014) | 365 | 30.7% | 304 | 28.3% |  |
| - NYHA class III | US CoreValve (Adams el al. 2014) | 365 | 4.7% | 304 | 4.6% |  |
| - NYHA class IV | US CoreValve (Adams el al. 2014) | 365 | 0 | 304 | 0.7% |  |
| - NYHA class I/II | PARTNER 1A (Mack et al. 2015) | 250 | 84.8% | 226 | 86.7% |  |
| - NYHA class III/IV | PARTNER 1A (Mack et al. 2015) | 250 | 15.2% | 226 | 13.3% |  |
| 24-month |  |  |  |  |  |  |
| - NYHA class I | US CoreValve (Reardon et al. 2015) | 252 | 62.7% | 190 | 57.9% | *P* = .66^†^ |
| - NYHA class II | US CoreValve (Reardon et al. 2015) | 252 | 29.4% | 190 | 32.6% |  |
| - NYHA class III | US CoreValve (Reardon et al. 2015) | 252 | 7.5% | 190 | 8.4% |  |
| - NYHA class IV | US CoreValve (Reardon et al. 2015) | 252 | 0.4% | 190 | 1.1% |  |
| 36-month |  |  |  |  |  |  |
| - NYHA class I | US CoreValve (Deeb et al. 2016) | 195 | 52.3% | 146 | 55.5% | *P* = .65^†^ |
| - NYHA class II | US CoreValve (Deeb et al. 2016) | 195 | 40.0% | 146 | 35.6% |  |
| - NYHA class III | US CoreValve (Deeb et al. 2016) | 195 | 6.2% | 146 | 8.2% |  |
| - NYHA class IV | US CoreValve (Deeb et al. 2016) | 195 | 1.5% | 146 | 0.7% |  |
| 60-month |  |  |  |  |  |  |
| - NYHA class I/II | PARTNER 1A (Mack et al. 2015) | 100 | 85.0% | 97 | 81.4% | *P* = .85^†^ |
| - NYHA class III/IV | PARTNER 1A (Mack et al. 2015) | 100 | 15.0% | 97 | 18.6% |  |
| Legend: n, number of patient; NYHA, New York Heart Association. * *P* value for the comparison of TAVI versus SAVR for the overall group of NYHA class I, II, III and IV. † Calculated by the authors of the current review. | | | | | | |
