# Supplementary material for: Transcatheter aortic valve implantation for aortic stenosis in high surgical risk patients: A systematic review and meta-analysis
Source: PLoS One. 2018 May 10;13(5):e0196877. doi: 10.1371/journal.pone.0196877 (PMC5944928; doi:10.1371/journal.pone.0196877)
Supplement: S16 Table — (DOCX) [file pone.0196877.s028.docx]

**S16 Table. Mean change in SF-12 from baseline: TAVI versus SAVR (operable at a high risk)**

| **Follow-up** | **Trial** | **TAVI** | | | | **SAVR** | | | | **Adjusted mean difference (95% CI)** | |  |
| --- | --- | --- | --- | --- | --- | --- | --- | --- | --- | --- | --- | --- |
|  |  | ***Transfemoral*** | | | | ***SAVR*** | | | | ***Physical summary*** | ***Mental summary*** |  |
|  |  | n | Physical summary | | Mental summary | | n | Physical summary | Mental summary |  |  |  |
| 1-month | PARTNER 1A (Reynolds et al. 2012) | 184 | 5.0 | | 4.3 | | 149 | 2.6 | -0.3 | 2.0 (0.1 to 3.9), *P* = .04 | 5.4 (3.1 to 7.7), *P* < .001 |  |
|  | US CoreValve (Arnold et al. 2015) | 186 | 5.4 | | 3.5 | | 137 | 0 | -2.9 | 4.9 (3.1 to 6.7), *P* < .001 | 6.1 (3.8 to 8.5), *P* < .001 |  |
| 6-month | PARTNER 1A (Reynolds et al. 2012) | 149 | 6.7 | | 5.1 | | 134 | 7.2 | 4.0 | -0.9 (-3.0 to 1.2), *P* = .41 | 1.2 (-1.0 to 3.5), *P* = .28 |  |
|  | US CoreValve (Arnold et al. 2015) | 210 | 6.3 | | 5.2 | | 159 | 6.8 | 2.7 | -0.3 (-2.1 to 1.4), *P* = .721 | 2.2 (0.3 to 4.1), *P* = .026 |  |
| 1-year | PARTNER 1A (Reynolds et al. 2012) | 187 | 6.3 | | 5.3 | | 147 | 6.1 | 4.7 | 0.41 (-2.8 to 2.0), *P* = .77 | 0.4 (-1.8 to 2.7), *P* = .69 |  |
|  | US CoreValve (Arnold et al. 2015) | 67 | 5.9 | | 4.8 | | 57 | 5.1 | 2.9 | 0.1 (-2.0 to 2.2), *P* = .927 | 0.8 (-1.3 to 3.0), *P* = .456 |  |
|  |  | ***Transapical*** | | | | | ***SAVR*** | | |  |  |  |
|  | PARTNER 1A (Reynolds et al. 2012) | n | Physical summary | | Mental summary | | n | Physical summary | Mental summary |  |  |  |
| 1-month |  | 76 | 2.8 | | -0.8 | | 61 | 0.5 | 1.7 | -5.8 (-17.9 to 6.4), *P* = .35 | 0.3 (-2.7 to 3.3), *P* = .85 |  |
| 6-month |  | 70 | 5.2 | | 3.3 | | 57 | 5.7 | 3.7 | -3.8 (-15.1 to 7.5), *P* = .51 | -3.3 (-6.7 to 0.0), *P* = .05 |  |
| 1-year |  | 66 | 7.1 | | 3.6 | | 58 | 4.5 | 3.9 | 6.1 (5.9 to 18.1), *P* = .32 | 0.2 (-3.5 to 3.8), *P* = .92 |  |
|  |  | ***Non-transfemoral*** | | | | | ***SAVR*** | | |  |  |  |
|  | US CoreValve (Arnold et al. 2015) | n | Physical summary | | Mental summary | | n | Physical summary | Mental summary |  |  |  |
| 1-month |  | 29 | 1.7 | | -2.8 | | 21 | -1.0 | 0.4 | 3.2 (-0.09 to 7.4), *P* = .126 | -0.1 (-5.4 to 5.1), *P* = .957 |  |
| 6-month |  | 38 | | 6.3 | 0.026 | | 32 | 3.4 | 2.8 | 0.1 (-0.35 to 3.7), *P* = .975 | -1.0 (-5.0 to 2.9), *P* = .609 |  |
| 1-year |  | 36 | | 6.6 | 0.023 | | 25 | 6.1 | 4.8 | 2.9 (-1.9 to 7.8), *P* = .237 | 1.3 (-3.7 to 6.3), *P* = .610 | |

Legend: 95% confidence intervals but not standard deviations were reported for the mean changes in the papers. CI, confidence interval; n, number of patients; SF-12, Short Form-12 General Health Survey.
