# Supplementary material for: Transcatheter aortic valve implantation for aortic stenosis in high surgical risk patients: A systematic review and meta-analysis
Source: PLoS One. 2018 May 10;13(5):e0196877. doi: 10.1371/journal.pone.0196877 (PMC5944928; doi:10.1371/journal.pone.0196877)
Supplement: S17 Table — (DOCX) [file pone.0196877.s029.docx]

**S17 Table. Incidence and severity of prosthesis-patient mismatch: TAVI versus SAVR (operable at a high risk)**

| **PARTNER 1A (Hahn et al. 2013)** | **TAVI** | | **SAVR** | | **Analysis** |
| --- | --- | --- | --- | --- | --- |
|  | **n. analysed** | **% of patients** | **n. analysed** | **% of patients** |  |
| 30-day | 259 |  | 201 |  | *P* = .0079 |
| - Insignificant |  | 58.3 |  | 43.8 |  |
| - Moderate |  | 27.8 |  | 38.3 |  |
| - Severe |  | 13.9 |  | 17.9 |  |
| 6-month | 217 |  | 156 |  | *P* = .0194 |
| - Insignificant |  | 53.9 |  | 39.1 |  |
| - Moderate |  | 30.0 |  | 39.7 |  |
| - Severe |  | 16.1 |  | 21.2 |  |
| 1-year | 203 |  | 142 |  | *P* = .0147 |
| - Insignificant |  | 51.2 |  | 35.9 |  |
| - Moderate |  | 26.6 |  | 34.5 |  |
| - Severe |  | 20.2 |  | 29.6 |  |
| 2-year | 134 |  | 102 |  | *P* = .0193 |
| - Insignificant |  | 47.0 |  | 29.4 |  |
| - Moderate |  | 33.6 |  | 48.0 |  |
| - Severe |  | 19.4 |  | 22.5 |  |
|  | | | | | |
